# Fighting the Microbe

**Published:** 1893-08-12

**Authors:** 


### Fighting the Microbe.

His Highness the Microbe is making his way. He has even entered into manufacture. Whole sets of surgical instruments are now fashioned for his especial benefit—that is to say, to counteract his baneful power.

Knives and needles, forceps and scissors—all those beautifully made little instruments that, like Hamlet, are but cruel to be kind—are now being forged entirely of steel, and when necessary to be jointed, are so hung together that they may be easily disjointed, and—boiled!

Boiling water and steam kill any germ, even the anthrax microbe, one of the most poisonous of the whole fell tribe; and the boiling of surgical instruments to kill the germs is now in favour with the medical profession. So surgical instrument makers, whose trade is one of the most highly skilled in this country, must keep abreast of the times.

And this is not all. Special sterilising apparatus is also prepared, wherein the deadly spores may be made to yield up their malignant life. And there is sterilised silk and sterilised cat-gut also for suture and ligature work, so that the manufacturers are fully arming the profession for their constant fray.

The name given to these instruments is aseptic—a title which, we imagine, will become as fully known as antiseptic in the days that are to come. And beautifully are they and their non-aseptic brothers forged. 'Mid all the clamour and the clangour of competition, and the wailings of lost or diminishing trade, London calmly holds the field for the manufacture of high-class surgical instruments.

Here, in some famous works under the shadow of Guy's, (Down Brothers, St. Thomas's Street, Borough) they are wrought out by hammer on the anvil in what at first sight might seem primitive style. Here is the little blacksmith's fire, and, here beside the fire, is the anvil. As simple as beating out a horse-shoe you say!

Just so, but like many simple things the forging is very difficult, and requires great skill. Watch the workman—or artist in steel one might almost call him—as with lighter and heavier strokes he fashions the delicate scalpel out of the thin bar of steel. Every blow answers its purpose and plays its part. And before the instrument leaves the forging room it is perfect, save for its shining polish or its merciful sharpness.

It is shaped out of the solid steel, and will receive no addition for its handle, save perhaps in some instances a coating of nickel. Some of the ordinary instruments are still made, for certain practitioners prefer handles of ebony, but as the influence of the microbe makes itself felt, the aseptic solid steel instruments appear to be the goods of the future.

"Where is he taking them?" you ask, as the artist in steel gathers up a dozen exquisite cataract knives, and leaving his forge to a colleague, marches upstairs.

"He is going to finish them off," answers the employer. "Every man here makes his instrument from beginning to end. He may work from designs, but so far as the actual manual labour is concerned it is his from start to finish."

No wonder the men are highly skilled. Here is a direct incentive to high-class work—also a means of tracing home the slightest flaw in such work. The worker is not a mere factory "hand"—an almost mechanical part of a huge machine, but an "artist" in one sense of the word—entrusted with the responsibility of executing delicate and skilful work from designs, and doing it to his own and his employer's satisfaction. He is paid "by the piece," of course, and excellent money he earns.

But before following him upstairs, let us look at the steel. Here it is in long, thin, brown bars, no fewer than six different kinds of it. There is a very hard steel, used chiefly for knives; then a steel not quite so hard used for bone instruments; thirdly, a steel again not so hard, but which would bend rather than break, and is used for what are called deformity instruments. Then there is scissors steel used as its name suggests for making scissors and also certain varieties of bone cutting instruments; fifthly, there is the "lithotrite" steel, the title of which suggests the class of instruments for which it is intended, and the chief quality of which is exceeding toughness rather than hardness; and finally there is Bessemer steel.

These different varieties suggest the wide scope of the work and also the skill required at the outset to select the most suitable steel for the instruments made. And truly the variety of instruments is astonishing. Thus, there are about seventy different kinds of forceps, and of one of these—viz., for the teeth—there are between fifty and sixty different patterns.

The instrument having been beaten out on the anvil, there comes the filing, the hardening—if necessary the sharpening—the tempering, and the polishing.

Held in a vice the instrument is filed smooth, to get rid of all roughnesses and irregularities; then it is hardened by being heated in a fire to a straw colour and plunged into water, or, in the case of a blunt instrument, into oil.

It is tempered in a gas flame, ground, "lapped" on a lead wheel with fine emery and oil—lapping being in fact a fine form of grinding—and finally polished with crocus, an exceedingly fine emery powder.

And what are the most saleable instruments of the present time?

We venture to say that ninety-nine persons out of a hundred would not guess aright. Knives, some one would reply. But that is hardly any answer, because there are so many different kinds of knives used for so many different purposes.

No, strange though it may seem, yet among the most saleable instruments are those for removing a morbid growth at the back of the nose, a complaint apparently very common with children. Some light upon prevalent maladies is thrown by visiting surgical instrument works!

Certain other things, of course, are also in pretty constant and large demand, and in high class works such as these the partners have to keep abreast of all the latest developments of surgery. Here, for instance, is the most recent antiseptic dressing—double zinc cyanide gauze and wool invented by Professor Lister, and the wood wool pulp from pine wood and impregnated with compounds of mercury.

Electricity, too, makes its influence widely felt, as well as the germ, and here are tiny electric lights for examining internal organs. Imagine a glow light thrust down your throat! Electric cauteries also are in vogue, but they have not everything their own way, for there is, further, a clever piece of apparatus—not electric—for maintaining the cauterising point at a high heat while the operation is being performed. "Up-to-date" might be written as the motto of such a factory as this.

What an immense variety of operations do these numerous instruments suggest! What a variety of means to relieve human suffering! Within these walls are gathered scores and scores of ideas wrought out into steel for the benefit of mankind. And it is not unworthy of note that in an age of machinery, skilled hand labour plays such an important part in preparing them for the practitioner.

